# Cell‐specific ablation in the testis: what have we learned?

**DOI:** 10.1111/andr.12107

**Published:** 2015-10-07

**Authors:** L. B. Smith, P. J. O'Shaughnessy, D. Rebourcet

**Affiliations:** ^1^ MRC Centre for Reproductive HealthUniversity of EdinburghThe Queen's Medical Research InstituteEdinburghUK; ^2^ College of MedicalVeterinary and Life SciencesInstitute of BiodiversityAnimal Health and Comparative MedicineUniversity of GlasgowGarscube CampusGlasgowUK

**Keywords:** animal models, germ cell transplantation, germ cells, gonadal development, Leydig cells, macrophages, paracrine factors, Sertoli cell, spermatogenesis, testis

## Abstract

Testicular development and function is the culmination of a complex process of autocrine, paracrine and endocrine interactions between multiple cell types. Dissecting this has classically involved the use of systemic treatments to perturb endocrine function, or more recently, transgenic models to knockout individual genes. However, targeting genes one at a time does not capture the more wide‐ranging role of each cell type in its entirety. An often overlooked, but extremely powerful approach to elucidate cellular function is the use of cell ablation strategies, specifically removing one cellular population and examining the resultant impacts on development and function. Cell ablation studies reveal a more holistic overview of cell–cell interactions. This not only identifies important roles for the ablated cell type, which warrant further downstream study, but also, and importantly, reveals functions within the tissue that occur completely independently of the ablated cell type. To date, cell ablation studies in the testis have specifically removed germ cells, Leydig cells, macrophages and recently Sertoli cells. These studies have provided great leaps in understanding not possible via other approaches; as such, cell ablation represents an essential component in the researchers’ tool‐kit, and should be viewed as a complement to the more mainstream approaches to advancing our understanding of testis biology. In this review, we summarise the cell ablation models used in the testis, and discuss what each of these have taught us about testis development and function.

## Introduction

Testicular development and function is the culmination of a complex process of autocrine, paracrine and endocrine interactions between multiple cell types. Early studies that focussed upon dissecting these interactions generally relied on cell culture systems or systemic interventions in vivo using chemicals or surgical hypophysectomy to suppress endocrine and downstream testicular function. While these studies have given valuable insight into testis biology they suffer the serious drawbacks that cells do not behave normally in culture, whereas systemic interventions cannot assign impacts or roles to specific cell types or genes. A more detailed understanding of testis development and function has arisen from the use of transgenic mouse models, ablating the function of single genes to determine their individual roles. The development of conditional gene targeting has further refined this, permitting ablation of specific gene function in single cell types, and indeed the combination of these reductionist approaches has identified many of the key genes essential for testis development and function. However, targeting genes one at a time does not capture the more wide‐ranging role of each cell type in its entirety. An often overlooked, but extremely powerful approach to elucidate cellular function is the use of cell ablation strategies, specifically removing one cellular population and examining the impacts to reveal the importance of that cell type for development and function.

**Figure 1:**
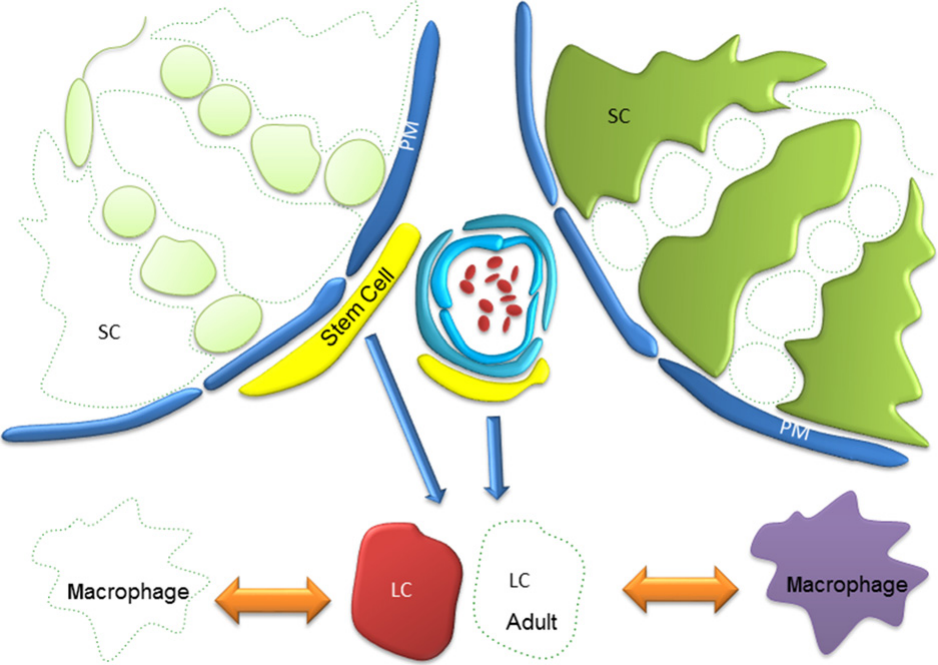
Schematic representation of the testis showing ablated cell populations. To date methodologies have been employed to selectively ablate germ cells, Leydig cells, Macrophages and recently Sertoli cells. By removing a single population and asking ‘what changes’ and ‘what remains the same?’, these studies have proven instrumental in defining our fundamental understanding of cell–cell communication in the testis, assigning specific roles to individual cell types whilst ruling out others. SC = Sertoli cell; PM = Peritubular Myoid cell; LC = Leydig cell; Stem cell = Leydig stem cell.

The major criticism levelled at cell ablation studies is the crudeness of the approach. It has been argued that removing an entire cell population, and thereby disrupting hundreds of signalling pathways simultaneously, provides little information regarding how the cell type in question influences other cellular populations. Whilst this is indeed true, and focussing on specific signalling pathways using this approach is flawed, evidence has demonstrated time and again that the power of cell ablation studies is to reveal a more holistic overview of cell–cell interactions. This not only identifies important roles for the ablated cell type, which warrant further downstream study, but also, and importantly, reveals functions within the tissue that occur completely independently of the ablated cell type. To date, cell ablation studies in the testis have specifically removed germ cells, Leydig cells, macrophages and recently Sertoli cells (Fig. 1). In this review, we will summarize the cell ablation models used in the testis, and discuss what each of these have taught us about testis development and function.

## Approaches to Cell Ablation

**Figure 2:**
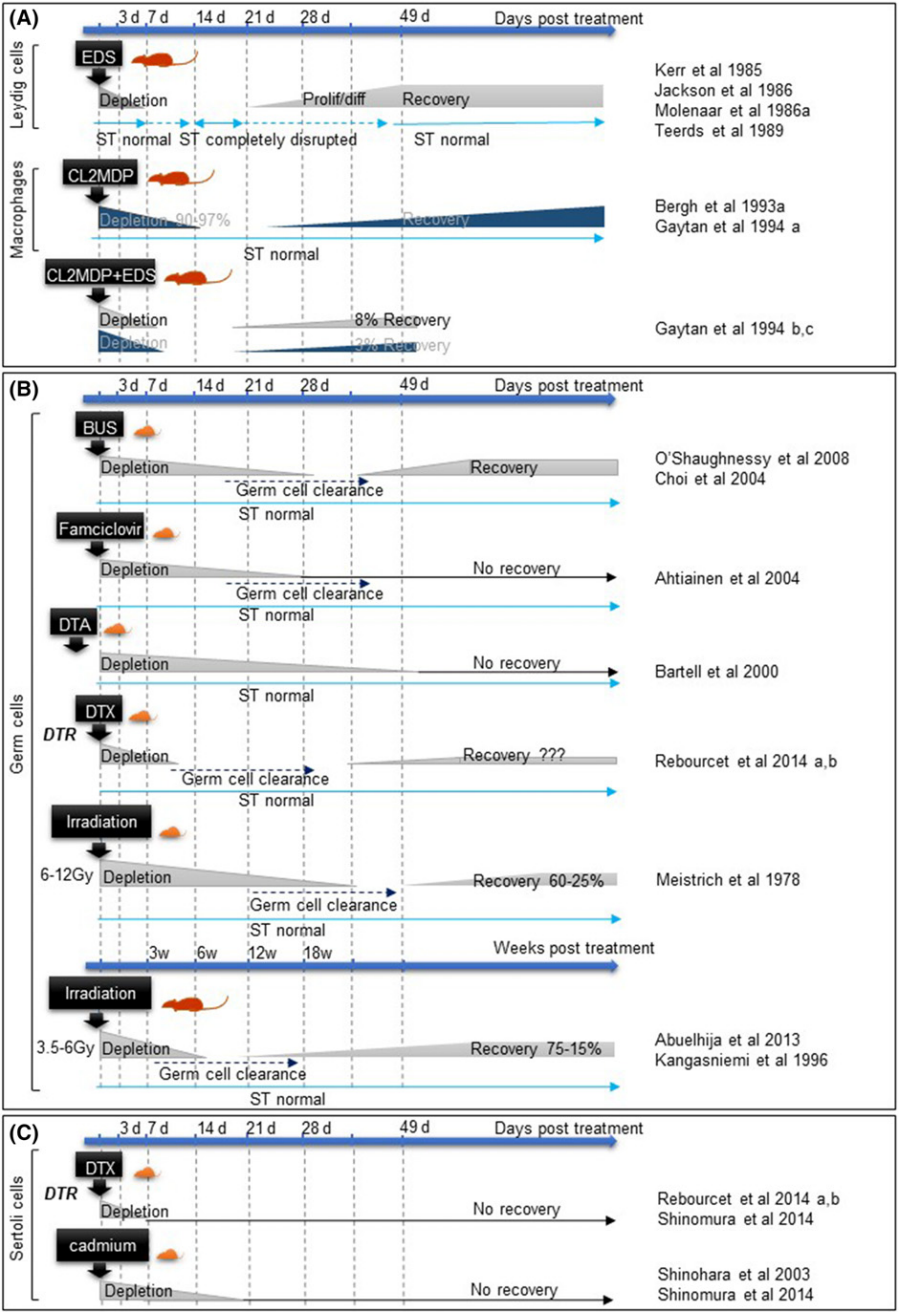
Schematic overview of depletion and recovery timings for each model of cell ablation. A schematic diagram comparing the rapidity of cell depletion and the duration of recovery days (d) after treatment, for each testicular cell type and method: (A) Leydig cells and macrophages when ablated independently or simultaneously; (B) Germ cells and (C) Sertoli cells. ST: seminiferous tubules, EDS: Ethylene dimethane sulphonate, CL2MDP: Liposome‐entrapped dichloromethylene diphosphonate, BUS: busulfan, DTA: active diphtheria fragment A, DTR: diphtheria receptor, DTX: diphtheria toxin.

**Figure 3:**
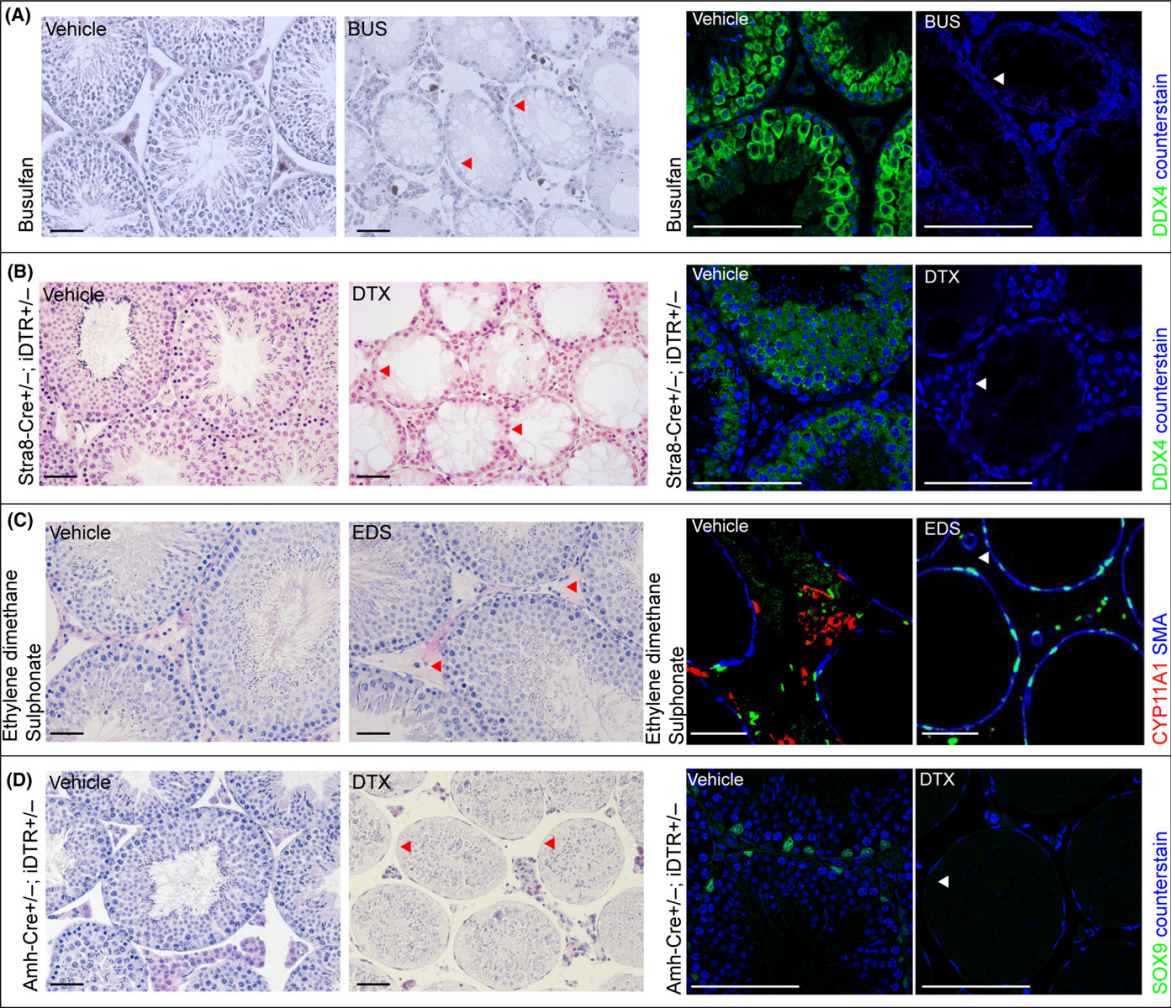
Cell‐specific ablation in the testis. Example of testis histology and immunolocalisation of cell‐specific markers following Germ cell, Leydig cell or Sertoli cell ablation respectively (indicated by arrowheads). (A) Germ cell ablation in mouse using busulfan (DDX4 staining to mark germ cells); (B) Germ cell ablation in Stra8‐Cre±;iDTR± mice using DTX injection (DDX4 staining to mark germ cells); (C) Leydig cell ablation in rat using EDS (CYP11A1 staining to mark Leydig cells). (D) Sertoli cell ablation in Amh‐Cre±;iDTR± mice using DTX injection (SOX9 staining to mark Sertoli cells); (scale bar: 100 μm).

A range of options are available to those wishing to selectively ablate a single population of cells from an organ, but there are several key features shared by all approaches. The choice of method to be used should: (i) be specific, only affect the target cell type; (ii) be precisely controllable, for example so that timing of cell death, etc., is known, (iii) have high efficiency of ablation, completely or almost completely remove the target population, (iv) act quickly, such that impacts can be directly attributed to cell loss; and (v) have minimal off‐target toxicity, such that other cell populations or organs are not affected. An overview of available options and their associated efficacy is shown in Fig. 2, and their impacts on testis architecture in Fig. 3.

Many models lacking a specific cell type have been generated as a serendipitous consequence of mutation or gene knockout. At the most basic level, knockout of Sry or Sox9 leads to sex reversal through failure of Sertoli cell specification, fundamentally demonstrating the requirement of Sertoli cells for masculinization [reviewed in (Kobayashi *et al*., 2005; Wilhelm *et al*., 2013)]. Within the developing testis knockout of other genes can lead to absence of specific cell types, for example knockout of PDGFR‐alpha (Brennan *et al*., 2003) impacts on foetal Leydig cell development, whereas knockout of desert hedgehog (DHH) (Clark *et al*., 2000), or COUPTFII (Qin *et al*., 2008) leads to absence of the adult Leydig cell population; W^v^ mutations of cKit lead to near germ‐free testes, through failure of primordial germ cell migration (De Franca *et al*., 1994), whereas mutations such as the cell‐specific loss of AR from Sertoli cells leads to loss of post‐meiotic germ cells (Chang *et al*., 2004; De Gendt *et al*., 2004). In each of these cases, however, it is challenging to assign any downstream phenotypic impact specifically to loss of gene function or to the wider effects of cell ablation. As such, and with the exception of the W/W^v^ mouse, few of these gene knockout models have been used widely as a model of cell ablation outside the context of their own gene function. To widen the scope of cell ablation to different genotypes and species several alternative approaches have been developed that induce cell loss of a specific testicular cell population without the requirement for gene knockout.

## Germ Cells

By far the most common approach to ablation of germ cells from the testis utilizes nascent chemotherapy agents such as busulfan (Myleran). The impact of busulfan on the spermatogenic cell population was first identified in the 1960s (Jackson *et al*., 1962), and its ability to apparently selectively remove the male germ cell population has been exploited widely in the past 50 years. This has been both in the pursuit of understanding about the role of germ cells in the testis, and also as a technical tool, important in preparation of recipients prior to germ cell transplantation (Brinster & Zimmermann, 1994; Brinster *et al*., 2003) [for review see (Brinster, 2002)], for fertility preservation and as a tool to investigate the spermatogonial stem cell niche. Busulfan is an alkylating agent that targets rapidly dividing mitotic spermatogonia resulting in apoptosis of this germ cell stage within 1 week, with a second wave of apoptosis in meiotic spermatocytes after 2 weeks (Choi *et al*., 2004) (Fig. 2). For these reasons, busulfan requires several weeks to completely ablate germ cells from the testis as the residual post‐mitotic stages complete spermatogenesis and are released into the seminiferous tubule lumen (Medrano *et al*., 2014) (Fig. 3).

Exposure to radiation will also cause mitotic cells to undergo apoptosis and has been used as a technique to study the effects of germ cell ablation (Fig. 2). At high doses radiation exposure in rat models can lead to infertility associated with a persistent failure to re‐establish spermatogenesis (Kangasniemi *et al*., 1996) and this has provided insight into how radiation‐induced infertility can occur, with direct relevance to cancer patients undergoing radiotherapy. For example reciprocal germ cell transplant studies (irradiated spermatogonia into non‐irradiated hosts or vice versa) reveal that, whereas germ cell loss is likely a direct result of impacts on germ cells, failure to re‐establish spermatogenesis is a somatic cell problem rather than any direct impact on spermatogonial stem cells (Zhang *et al*., 2007), suggesting radiation acts on both germ cells and somatic cells simultaneously.

The ability of very defined doses of radiation to selectively remove the germ cell population has also been explored as a method to generate recipient animals for germ cell transplants, especially in scenarios where genetic mutants lacking germ cells (e.g. W/W^v^) mice are unavailable or inappropriate (e.g. difficult to breed, immunocompatibility issues with donor cells, use of other species) (Zhang *et al*., 2006). Even within mice, the removal of germ cells using busulfan is a strain‐dependent balance between the required toxicity to kill sufficient germ cells and the sensitivity of the animal to the wider toxicological effects of the drug, particularly on haematopoiesis (Meistrich *et al*., 1978; Abuelhija *et al*., 2013). In this context, radiation provides a useful alternative to busulfan; the fact that it has not been taken up widely probably reflects the general move of researchers away from use of radiation wherever possible and the ready availability and convenience of a single busulfan injection which requires significantly less specific training, infrastructure or administrative oversight.

An alternative model to either busulfan or radiation utilises a unique transgenic approach, expressing the Herpes Simplex virus Thymidine Kinase (TK) transgene from an inhibin‐alpha promoter, which directs expression to Leydig and Sertoli cells, but not spermatogonia (Ahtiainen *et al*., 2004). The TK enzyme phosphorylates analogue nucleosides such as famciclovir, which are then incorporated into replicating DNA, acting as chain terminators and leading to eventual cell death. In the Inha/TK mouse, mitotically quiescent Sertoli and Leydig cells are resistant to the effects of TK, whereas the rapidly dividing spermatogonia cells are susceptible, probably through a bystander effect mediated by the gap junctions with Sertoli cells (Ahtiainen *et al*., 2004). As with busulfan, the ontogeny of cell death is gradual, taking 4 weeks to remove all germ cell populations through selective ablation of spermatogonia followed by natural development and sloughing of later germ cell stages until the testis is devoid of germ cells (Fig. 2). Whilst time to germ cell clearance is long, the authors show that a 3‐day treatment is sufficient to completely ablate all germ cell populations, and they observe no recovery 3 months post treatment. This could either be attributed to complete cell death of all spermatogonial stem cells, or impacts on Sertoli cells that prevent recovery of spermatogenesis. (Ahtiainen *et al*., 2004).

Diphtheria toxin (DTX) has also been used in several models of germ cell ablation. In one model expression of the diphtheria toxin A‐chain gene is directed to the male germline by fusion to 1 KB of the five prime flanking DNA of the rat male‐germ cell‐specific histone H1t gene (Bartell *et al*., 2000). The H1t promoter fragment drives cell‐specific expression of the diphtheria toxin A fragment in pre‐spermatogonia. This results in widespread cell death, with just a few germ cells reaching the pachytene spermatocyte stage, and by 13 weeks of age the testis is almost completely devoid of germ cells (Fig. 2). In contrast, females are completely fertile (Bartell *et al*., 2000). Although the testes in these animals are extremely small, seminal vesicle weights are normal, suggesting that loss of germ cells does not impact androgen production.

In contrast to direct expression of the DTX A‐chain inside germ cells, other transgenic approaches have been used which exploit the natural resistance of mice to DTX. In primates, DTX enters cells by binding to a cognate HBEGF receptor (also known as Diphtheria Toxin Receptor – DTR) and it is this internalisation followed by cleavage of the A‐chain, which leads to cell death. In mice, DTX has low affinity for mHBEGF but transgenic expression of an inducible primate iDTR within mouse germ cells confers sensitivity to injected recombinant DTX. Breeding of iDTR to a germ cell Cre Recombinase line (Stra8‐Cre) (Sadate‐Ngatchou *et al*., 2008) results in expression of iDTR in male germ cells from postnatal day (pnd) 3 onwards (Rebourcet *et al*., 2014b). These animals show normal fertility but germ cell‐specific cell death can be induced at any time of choosing through injection of DTX. Cell death is rapid, with apoptosis induced within 24 h and near complete clearance of germ cells from the seminiferous tubules within 7 days of a single injection (Fig. 2 and 3). To date this approach combines the fastest clearance of all germ cells with the lowest off‐target toxicity although this approach does rely on the presence of multiple transgenes, which is impractical in most contexts. Furthermore, because of the variable nature of the Cre used (Bao *et al*., 2013), a small number (<1%) of spermatogonia fail to express the receptor and thus remain in the testis. However, the fact that these residual cells are able to repopulate the testis to produce complete spermatogenesis, demonstrates there is no long‐term impact of germ cell ablation on testis function using this approach (Rebourcet *et al*., 2014a,b).

**Figure 4:**
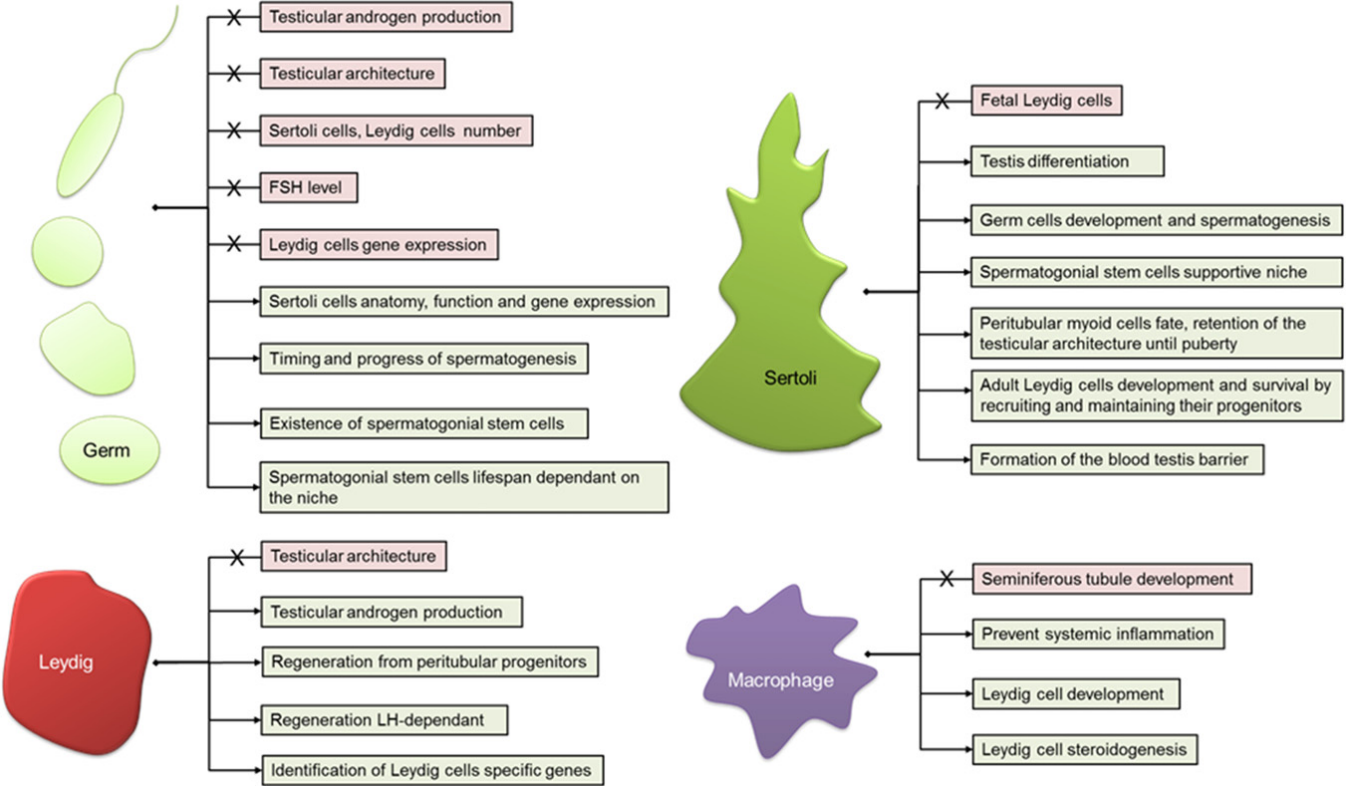
What have cell‐ablation models taught us? Overall testis differentiation, somatic cell development and testicular endocrine function is independent of Germ cell regulation. However, germ cell‐ablation models have highlighted the intricate cross talk between germ cells and Sertoli cells. germ cell ablation combined with transplantation studies have also shown that germ cells control the timing and the progression of spermatogenesis, although the lifespan of the spermatogonial stem cells and consequently spermatogenesis depends on the somatic cell niche. Leydig cells are the source of androgens, essential for spermatogenesis. When ablated, Leydig cells are able to regenerate from peritubular progenitors under regulation from Luteinising hormone (LH) and other testicular secreted factors. Cell ablation studies have permitted the complete dissection of this regeneration process, which is believed to recapitulate normal adult Leydig cell development in most respects. Leydig cell‐ablation studies have also aided the identification of Leydig cell‐specific transcripts. The testis is an immune privileged organ. Macrophage ablation studies have shown that these cells are not essential to overall seminiferous tubules development. They prevent local systemic inflammation engendered by Leydig cell death. Macrophages and Leydig cells share an intricate physical and physiological relationship; macrophage ablation has revealed that this relationship is essential for Leydig cell development and regeneration. Sertoli cells orchestrate testis differentiation. In prenatal and pre‐pubertal life they are essential for the development and maintenance of testicular architecture. Sertoli cells are crucial components of the spermatogonial stem cell niche, permitting germ cell development and differentiation (spermatogenesis). Sertoli cell‐ablation models have shown that, following their initial differentiation, Fetal Leydig cells are no longer dependent on Sertoli cells. In contrast the development and survival of the adult Leydig cell population is fundamentally dependent upon Sertoli cells.

Germ cell ablation has significantly enhanced our understanding of testis development and function (Fig. 4). The most striking observation arising from germ cell loss is that the testis can form a normal architecture in the complete absence of germ cells. This suggests that germ cells play little role in the architecture of the developing testis. Indeed W/W^v^ mice, which lack germ cells, have been used to demonstrate that germ cells play no role in the increase in Sertoli cell numbers observed in cases of hypothyroidism (Saxena *et al*., 2002), strongly suggesting that hypothyroidism acts through a direct effect on Sertoli cells [reviewed in (Holsberger & Cooke, 2005)]. This apparent lack of impact on testicular architecture is also observed if germ cells are ablated in adulthood (Fig. 3), with no effect seen on either Sertoli cell (De Franca *et al*., 1994; O'Shaughnessy *et al*., 2003) or Leydig cell numbers (O'Shaughnessy *et al*., 2003) and retention of general testicular architecture. Further to this, ablation of germ cells in adulthood does not impact on Leydig cell gene expression (O'Shaughnessy *et al*., 2008a) or androgen concentrations, with no change observed in either intratesticular testosterone (O'Shaughnessy *et al*., 2008a) or circulating testosterone concentrations (Gomes *et al*., 1973; Morris *et al*., 1987; De Franca *et al*., 1994), suggesting that germ cells play little role in control of testicular androgen production. In contrast to the lack of effect on Leydig cells, germ cell loss significantly impacts Sertoli cell anatomy (a significant reduction in smooth endoplasmic reticulum (De Franca *et al*., 1994)) and function (although not Sertoli cell number). By following the progressive nature of germ cell loss after busulfan treatment, it is clear that the Sertoli cells are most influenced by post‐meiotic germ cells, with significant changes in Sertoli cell gene transcript levels apparent only when these germ cells are lost from the seminiferous epithelium, ~15–30 days post busulfan injection. Whilst there are also gene expression changes at earlier time points, these are not as widespread suggesting spermatogonia and spermatocytes exert less influence over Sertoli cell gene expression (O'Shaughnessy *et al*., 2008b).

Perhaps one of the most intriguing observations is the relationship between germ cells and circulating follicle stimulating hormone (FSH) levels. The current paradigm dictates that FSH levels will increase in response to a reduction in germ cell number, via antagonism of Sertoli cell production of InhibinB, which acts in a negative feedback loop in the pituitary [reviewed in (Meachem *et al*., 2001)]. Whilst this concept is supported by the observation of a significant increase in circulating FSH concentrations following busulfan‐induced germ cell loss in the rat (Gomes *et al*., 1973), and in the mutant W/W^v^ mouse (De Franca *et al*., 1994), this is not observed in wild‐type mice where in contrast, complete loss of germ cells either through busulfan injection (O'Shaughnessy *et al*., 2008a) or diphtheria toxin injection (Rebourcet, O'Shaughnessy, Smith, unpublished data) results in no increase in FSH levels whatsoever. It is difficult to reconcile these observations, although the fact that FSH levels *are* significantly increased in a mouse model of Sertoli cell ablation (Rebourcet *et al*., 2014a) suggests that the primary insult may be to the Sertoli cells. It could therefore be speculated that either the rat germ cells influence Sertoli cells to a greater extent than in the mouse, or alternatively, that rat Sertoli cells are more sensitive to perturbation by busulfan.

In addition to advancing our understanding regarding the control of testis development and cell‐cell interactions, germ cell ablation, when combined with subsequent germ cell transplantation has also provided significant understanding of germ cell development. In a groundbreaking study published in 1994, Brinster and Zimmermann showed that spermatogenesis was possible following germ cell transplantation in immunocompatable mice (Brinster & Zimmermann, 1994). Key to the success of the study was the use of mice lacking germ cells, with both W/W^v^ mice and busulfan‐treated mice successfully used. This revolutionary approach opened up the possibility of supporting spermatogenesis in response to adverse clinical conditions, for example fertility preservation in cancer patients. Along the way, however, use of germ cell ablation and transplantation has also enlightened our understanding of germ cell development and function, for example permitting the characterization of the pattern and kinetics of donor‐derived spermatogonial stem cell colonisation. This includes the observation that Sertoli cells recognise and permit retrograde spermatogonial transit through the tight junctions of the blood–testis barrier (Nagano *et al*., 1999). Through this approach it has also been shown that cross‐species transplantation of rat spermatogonia into mice results in full ‘rat’ spermatogenesis (Clouthier *et al*., 1996), with the germ cells taking 52–53 days to fully mature, rather than the normal 35 days of mouse spermatogenesis (Franca *et al*., 1998). This means that it is the germ cells, rather than Sertoli cells, which control the timing and progress of spermatogenesis.

Identifying the stem cells amongst the spermatogonia lining the basement membrane of the seminiferous tubules using morphological parameters has proven challenging. Germ cell ablation and transplantation has been used to estimate the number of spermatogonial stem cells through counting of individual colonies of expanding spermatogonia marked with B‐galactosidase. Initial estimates using transplant into adult mice has produced an estimate of ~0.002% of total spermatogonia number (Nagano *et al*., 1999), however, transplant into younger recipients in which the blood–testis barrier (BTB) had yet to form increases colony number 10‐fold suggesting the initial estimates were hindered by the ability for stem cells to cross the BTB in a retrograde direction and colonise available niches (Shinohara *et al*., 2001). The functional lifespan of spermatogonial stem cells has also been established using cell ablation and transplantation. These studies show that, whilst under normal circumstances stem cell number and proliferative capacity drops off with age, stem cells retain their proliferative capacity if transplanted into younger mice. Serial transplantation experiments demonstrate that this remains the case for more than 2 years in vivo in the mouse, significantly longer than the normal breeding window (Ogawa *et al*., 2003; Ryu *et al*., 2006), and highlighting the degeneration of the niche as the limiting factor in lifelong spermatogenesis.

Germ cell ablation and transplantation studies have also been used to establish when germline stem cells acquire spermatogonial stem cell activity. Ohta *et al*., (2004) have shown that gonocytes collected from 13.5 days post coitum (dpc) embryos or later stages can colonise seminiferous tubules. Moreover, when transplanted into younger, 5–10 day old recipients, not only primordial germ cells from all foetal ages, but also epiblasts from 6.5 dpc embryos could colonise and go on to produce spermatozoa (Chuma *et al*., 2005). Taking this to extreme levels, it is now possible (but challenging and with poor efficiency) to generate PGC‐like cells from ES cells and transplant these into host testes to produce spermatids and live mice through assisted reproduction techniques (Hayashi *et al*., 2011).

As discussed above, the development of knockout mouse models has revolutionised genetics and our ability to study complex biochemical and physiological mechanisms. Until the more recent development of conditional knockout technologies, however, one of the key issues when examining the role of genes in knockout animals was the inability to assign gene function to a specific cell type if the gene is expressed in several cells. Germ cell ablation and transplantation using mutant donor germ cells or mutant recipients has been successfully used to dissect the Steel/cKit tyrosine receptor system, mutations in which lead to a block in spermatogenesis. In the testes of mice homozygous for mutations in either cKit (W/W^v^) (germ cell) or the kit ligand Steel factor (Sl/SLd) (Sertoli cell) the seminiferous tubules lack almost all germ cells (Ogawa *et al*., 2000). However, transplantation of wild‐type germ cells into W/W^v^ mice restores fertility in W/W^v^ mutants, whereas transplantation of spermatogonial stem cells from Sl/Sld males into W/W^v^ mice also restores fertility (Ogawa *et al*., 2000), both by overcoming the cell‐specific impacts of their respective gene loss. As the expression of the ligand and the receptor differs (ligand‐SC and receptor‐GC), the germ cell loss defect in both mutants originate either from the precursor cells (W/W^v^) or from the microenvironment (Sl/SLd). Therefore, the outcome after transplantation of green fluorescent protein (GFP) tagged germ cells into the seminiferous tubules of Sl/SLd mice resulted in colonisation but no differentiation of the GFP germ cells, which remain as spermatogonia. In contrast, when they are re‐transplanted into W/W^v^ mice complete spermatogenesis was achieved (Ohta *et al*., 2000).

This initial proof of principle for assigning gene roles to either germ cell or somatic cell populations has been expanded to other genes, showing, for example that transplantation of spermatogonia from juvenile spermatogonial depletion *jsd*‐mutant mice, which show an arrest of spermatogenesis after one round of meiosis, into wild‐type host mice fails to restore fertility. In contrast, transplant of wild‐type germ cells into a *jsd* host leads to full spermatogenesis, confirming that the *jsd* mutation is an intrinsic problem with the germ cells, and not the somatic environment (Boettger‐Tong *et al*., 2000). In a similar approach, transplantation of germ cells from estrogen receptor‐alpha (αERKO) knockout mice, which are infertile, into germ cell‐depleted hosts leads to normal spermatogenesis, showing it is the somatic environment underpinning this particular infertility phenotype (Mahato *et al*., 2000). Similarly, germ cells from infertile testicular feminized (*Tfm*) mice (which have a null mutation in the androgen receptor (*Ar*)) are able to go on to produce spermatozoa once transplanted into a wild‐type host mouse (Johnston *et al*., 2001). This approach has been widely exploited with investigations into the role of genes such as *Etv5* (Morrow *et al*., 2007) *Claudin11* and *Rac1* (Takashima *et al*., 2011) and factors such as vitamin‐A (McLean *et al*., 2002) in the regulation of spermatogonial stem cell niche populations [reviewed in (Kanatsu‐Shinohara & Shinohara, 2013)].

To a large extent the development of conditional gene targeting and the wide availability of germ cell expressing Cre recombinase lines [reviewed in (Smith, 2011)] has superseded the requirement for such transplantation experiments to establish gene function. In fact the protocol has become so mainstream that today it is largely exploited for what it can do as a technology, for example xenogenic germ cell development for endangered species [which is itself not without problems (reviewed in (Paris *et al*., 2004)], or as an approach to generate transgenic domesticated animals (Zeng *et al*., 2013). Most recently germ cell ablation and transplantation has been associated with a drive towards the generation of artificial gametes (gametes generated by manipulation of their progenitors or of somatic cells) (Hendriks *et al*., 2015a), which raises significant moral and ethical questions that need to be debated by society in the coming years (Hendriks *et al*., 2015b).

## Leydig Cells

The Leydig cells are the source of androgen production by the testis and these androgens are essential for male phenotypic differentiation, fertility and libido. It is clear that in eutherian mammals there are two populations of Leydig cells which arise sequentially during development (Roosen‐Runge & Anderson, 1959; Lording & De Kretser, 1972). The foetal population of cells develops soon after testis differentiation and is essential for foetal synthesis of androgen and INSL3 which ensure masculinization of the foetus and testis descent (Nef & Parada, 1999). In all mammals so far studied a second, adult population of Leydig cells starts to develop in the pre‐pubertal period [around days 7–10 in mice (Baker *et al*., 1999; Nef *et al*., 2000)]. This adult population of cells secretes androgens essential for male phenotypic and behavioural development and puberty and maintains fertility in the adult. The adult Leydig cells are completely dependent on LH support for both development and adult function; in the absence of LH few adult Leydig cells develop and circulating testosterone levels are barely detectable (O'Shaughnessy *et al*., 1998; Baker & O'Shaughnessy, 2001; Lei *et al*., 2001; Zhang *et al*., 2001). In contrast, the foetal Leydig cells in rodents appear to function largely independently of hormonal support (El Gehani *et al*., 1998; O'Shaughnessy *et al*., 1998; Baker *et al*., 1999; Lei *et al*., 2001; Zhang *et al*., 2006) although in other species LH (or hCG in humans) is required for foetal androgen production (O'Shaughnessy & Fowler, 2011).

Leydig cell ablation has proven incredibly informative in improving our understanding of testis development and function (Fig. 4). Studies of Leydig cell ablation started largely by chance through observations of the effects of ethane dimethane sulphonate (EDS) in the rat. In the 1960s Harold Jackson and colleagues were examining anti‐spermatogenic agents as potential regulators of male fertility [see (Jackson, 1970)]. EDS is structurally very similar to busulfan and a single dose was shown, like busulfan, to cause disruption of the seminiferous epithelium over a similar time scale (Jackson, 1970). One significant difference, however, was that treatment with EDS led to a marked loss in weight of the prostate and seminal vesicles indicating loss of testosterone and a change in Leydig cell function (Jackson, 1970; Jackson & Jackson, 1984). Subsequently, in the mid‐1980s it was shown that EDS acts by causing rapid ablation of the Leydig cells within 48 h of a single injection (Kerr *et al*., 1985; Molenaar *et al*., 1985; Morris *et al*., 1986) (Fig. 2). Other effects of EDS on the testis (e.g. loss of spermatogenesis) were shown to be due largely to the consequences of androgen withdrawal although some effects on Sertoli cell and peritubular myoid cell function have been reported (Verhoeven *et al*., 1989; Roberts & Griswold, 1990). EDS is a glutathione‐dependent alkylating agent (Kelce & Zirkin, 1993; Morris, 1996), which induces apoptosis in Leydig cells through activation of the Fas system (Taylor *et al*., 1999; Kim *et al*., 2000) and possible upregulation of N‐myc downstream‐regulated gene 2 (NDRG2) (Li *et al*., 2012). It is not clear, however, how EDS acts to induce the apoptotic pathway or why EDS is specific to Leydig cells. There is also strong species specificity to the actions of EDS on Leydig cells with little effect of the toxicant seen in a number of species including the mouse, dog and monkey, whereas others such as the rat, guinea pig and frog show high sensitivity (Morris, 1996). This may provide a further clue to the specificity of the toxicant as it has been shown that in rats the Leydig cells show specific expression of a number of metabolic enzymes which act to metabolise xenotoxicants (O'Shaughnessy *et al*., 2014). It is possible that one of these enzymes may serve to activate EDS and species specificity may arise from variability in substrate specificity or enzyme isoforms expressed.

In the years since its actions were first identified EDS has proved an extremely valuable tool in our efforts to understand Leydig cell biology. Its usefulness is only limited by its failure to act in mice (except at very high doses which will probably have major systemic effects (Li *et al*., 2012)), which means it cannot be used in conjunction with the numerous gene knockout models available in that species. Much of the interest in EDS has centred on the key observation that the Leydig cell population in the adult will regenerate in 3–6 weeks following ablation (Kerr *et al*., 1985; Molenaar *et al*., 1986). This is important because it shows that Leydig cell stem cells persist in the adult testis and that the potential exists to regenerate Leydig cells in situations such as ageing where Leydig number and/or activity is reduced (Chen *et al*., 1996). In addition, Leydig cell re‐generation after EDS appears to closely resemble normal development of the adult Leydig cell population thereby providing a model system for study of this process (Teerds & Rijntjes, 2007; Guo *et al*., 2013). The advantages of this model over normal development are that the interstitium is clear of Leydig cells and so there can be no confusion between developing and mature Leydig cells, other developmental events in the testis are not occurring simultaneously and hormone levels can be more easily manipulated in the more mature animals.

Study of Leydig cell re‐generation after EDS has identified the origin of the differentiating Leydig cells, the role of gonadotrophins and has started to identify other factors which may be involved in the process. The identity of the adult Leydig cell precursor population has been contentious for a number of years with pericytes, peritubular cells, fibroblasts, and endothelial cells all suggested (Jackson *et al*., 1986; Kerr *et al*., 1987; Teerds *et al*., 1990; Davidoff *et al*., 2004; O'Shaughnessy *et al*., 2008b; Stanley *et al*., 2012). Recent data indicates, however, that the major source of adult Leydig cells after EDS treatment are peritubular cells (O'Shaughnessy *et al*., 2008b; Stanley *et al*., 2012) although a contribution from other sources cannot be ruled out. The role of gonadotrophins in Leydig cell regeneration was established in early EDS studies using hypophysectomised rats or testosterone implants. This work showed that Leydig cell regeneration after EDS is dependent on LH (Molenaar *et al*., 1986; Sriraman *et al*., 2003) although some early proliferation and differentiation appears to occur independent of LH (Teerds *et al*., 1989). Subsequent studies have shown that similar control mechanisms regulate normal adult Leydig cell development at puberty (Clark *et al*., 2000; Gnessi *et al*., 2000; Baker & O'Shaughnessy, 2001; Zhang *et al*., 2001, 2004; Baker *et al*., 2003). Other factors such as DHH, PDGF and anti‐Müllerian hormone (AMH) have been implicated in the development of Leydig cells both during development and re‐generation (Salva *et al*., 2004; Josso *et al*., 2006; O'Shaughnessy *et al*., 2008b) and further studies on EDS regeneration have also suggested that nerve growth factor (NGF) and Sertoli cells are involved in Leydig cell proliferation, differentiation and survival (Yan *et al*., 2000; Zhang *et al*., 2013).

While progress has clearly been made in our understanding of adult Leydig cell development and regeneration using EDS, questions remain to be answered. For example data from mutant and knockout mice make it clear that LH is essential for Leydig cell differentiation but do changes in LH concentrations explain the onset and control of Leydig cell development? LH levels certainly rise after EDS treatment (Molenaar *et al*., 1986) and treatment of normal adult rats with LH or hCG for up to 5 weeks is reported to stimulate an increase in Leydig cell number (Christensen & Peacock, 1980; Teerds *et al*., 1988; Mendis‐Handagama *et al*., 1998). Ablation of the germ cell population at the same time as EDS treatment, however, has been shown to cause more rapid regeneration of the Leydig cells without further affecting LH levels (Molenaar *et al*., 1986; Risbridger *et al*., 1987). It is also not clear that circulating LH levels rise prior to the start of normal adult Leydig cell development (Selmanoff *et al*., 1977; Michael *et al*., 1980; Jean‐Faucher *et al*., 1983; Murphy *et al*., 1994). Finally, we have shown that Sertoli cell ablation prevents normal Leydig cell development (Rebourcet *et al*., 2014b) (see also section 6) indicating that the Sertoli cells must be involved in this process. As suggested above, understanding what regulates Leydig cell development might allow controlled regeneration of Leydig cells in circumstances where Leydig cell numbers and/or activity are reduced. This may be particularly relevant to ageing (Beattie *et al*., 2015) as Leydig cells which regenerate in aged rats after EDS treatment have similar activity to Leydig cells from young rats and they are able to maintain serum testosterone levels in the aged rats at the same level as those in young rats (Chen *et al*., 1996).

Leydig cell ablation by EDS causes a marked reduction in intra‐testicular androgen levels and this technique has been used to investigate the role of androgens in the testis, particularly with reference to Sertoli cell function and spermatogenesis. Other techniques such as hypophysectomy or treatment with GnRH agonists or androgen receptor antagonists will have similar effects, however, and so data coming from EDS experiments are not unique in that respect. One of the more interesting observations using EDS was made in 1988 and showed that spermatogenesis can largely be maintained after EDS if the animal is treated with sufficient androgen (Sharpe *et al*., 1988). This implies that androgen is the only Leydig cell product that is essential for spermatogenesis although a repeat of this study with more up‐to‐date and sensitive measures of spermatogenesis and Sertoli cell activity would appear to be warranted.

The ability to ablate a specific cell type in a tissue should allow the identification of transcripts or proteins, which are specific to that cell type, or that rely on that cell type. This technique has been used, for example to show that expression and activity of the steroidogenic enzymes, with the exception of HSD17B3, is limited to the Leydig cells (Murphy & O'Shaughnessy, 1992; O'Shaughnessy *et al*., 2008b). More recently, arrays have been used in two independent studies to identify a comprehensive set of Leydig cell‐specific transcripts in the EDS rat model (O'Shaughnessy *et al*., 2014; Zhang *et al*., 2015). In both studies transcripts were identified that showed markedly reduced expression following Leydig cell ablation and in each case this included most of the known Leydig cell‐specific transcripts. Perhaps surprisingly, however, there are quite marked differences in the lists of Leydig cell transcripts identified in the two studies. For example of the 67 known genes identified by Zhang and colleagues (Zhang *et al*., 2015) as Leydig cell exclusive only 23 are also identified by O'Shaughnessy and colleagues (O'Shaughnessy *et al*., 2014). There are technical differences between the studies (e.g. the arrays used are different) but, perhaps more pertinent, the models are also slightly different. Zhang and colleagues (Zhang *et al*., 2015) used a ‘standard’ EDS model (normal adult rats treated once with EDS), whereas O'Shaughnessy and colleagues (O'Shaughnessy *et al*., 2014) used a germ cell‐free rat model. The rational for using germ cell‐free animals being that the reduction in androgen levels caused by EDS ablation of Leydig cells will cause germ cell apoptosis (Kerr *et al*., 1993) and, therefore, significant loss of germ cell‐specific transcripts. This is turn may complicate identification of transcripts lost through direct ablation of the Leydig cells. Despite differences between studies, however, the approach to identifying cell‐specific transcripts through cell ablation holds significant promise, and further studies should resolve differences in results so far published.

## Macrophages

The resident testicular macrophages (characterised as CD68−, CD163+; ‘ED2’) form the largest population of immune cells in the testis, and form a close physical and physiological relationship with Leydig cells in the testicular interstitium [reviewed in (Winnall & Hedger, 2013)], with approximately one resident macrophage for every four Leydig cells (Bergh, 1985). Under non‐inflammatory conditions, this population undergoes natural turnover through a slow infiltration of circulating (CD68+, CD163− ‘ED1’) monocytes/macrophages followed by a local conversion into the ED2 resident form (Sauter *et al*., 2014).

Macrophage ablation has significantly enhanced our understanding of cell–cell communication in the testis (Fig. 4). In a series of elegant studies in the rat, the testicular function of the resident macrophage population has been investigated using injection of liposome‐entrapped dichloromethylene diphosphonate into the testicular interstitium to ablate the macrophages (Bergh *et al*., 1993a). This treatment regimen in the adult reduces macrophage number in the injected testis by 90% 7 days post injection (Fig. 2). Results from these studies show that macrophages may play a previously unknown role in support of Leydig cell development and function as well as secreting factors that inhibit hCG‐induced testicular inflammation (Bergh *et al*., 1993b). During pre‐pubertal development, macrophage ablation results in an arrest in the size of the Leydig cell population until day 35, when macrophage repopulation permits ongoing Leydig cell development. Furthermore, the authors have demonstrated that, whereas exogenous hCG treatment for 6 days from day 21 results in a seven‐fold increase in numbers of Leydig cells, no increase in Leydig cell number is observed in the contralateral, macrophage‐ablated testis (Gaytan *et al*., 1994a), an observation confirmed in hypophysectomised and macrophage‐depleted rats treated with hCG (Gaytan *et al*., 1995c). Importantly, in this study, treatment with FSH increased testicular weight and seminiferous tubule diameter independently of macrophage ablation, demonstrating that macrophages are dispensable for this aspect of testis function (Gaytan *et al*., 1995c). This first indication of an essential role for testicular macrophages in the development of the Leydig cell population has been examined further using the EDS model of induced Leydig cell ablation and regeneration, with or without concurrent macrophage ablation. Results from this study showed that macrophages are essential for regeneration of the Leydig cell population after EDS, with the size of the population proportional to the number of macrophages, hinting at a physical, numerical and physiological relationship between the two populations (Gaytan *et al*., 1994b,c). In a contrasting study, the role of macrophages in removal of apoptotic cells has also been investigated. When Leydig cell ablation was carried out 10 days after macrophage ablation there was a resultant influx of inflammatory lymphocytes and monocytes, not seen in the contralateral macrophage‐containing testis. This suggests that resident macrophages may act to prevent a systemic inflammatory response to localised cell death in the testis (Gaytan *et al*., 1995b).

Unilateral macrophage ablation in the adult testis results in increased LH, circulating testosterone and intra‐testicular testosterone concentrations, with intra‐testicular testosterone concentrations higher in the macrophage‐retaining testis. This suggests that, although macrophages are essential for the development of the Leydig cell population, once established the macrophages enhance Leydig cell steroidogenesis, but are not essential (Gaytan *et al*., 1996). Indeed, treatment with hCG following bilateral ablation of the macrophage population leads to a 3.5‐fold increase in serum testosterone, compared with an 8.6‐fold increase in intact controls (Gaytan *et al*., 1995a). A number of studies have also focussed on deciphering the mechanism involved in macrophage regulation of Leydig cell function. Using macrophage‐conditioned media a lipophilic factor has been identified (Hutson *et al*., 1996) which appears to alter Leydig cell androgen synthesis through a StAR‐independent pathway (Lukyanenko *et al*., 1998).

An alternative method of ablating the macrophage population in adulthood, – chronic 6‐week treatment with anti‐CSF1R antibody (which prevents development of replacement macrophages) – causes a small but significant reduction in testis weight (Sauter *et al*., 2014). In this study, however, circulating LH and testosterone were unaffected, perhaps reflecting the development of a compensatory mechanism over the time course of treatment in contrast to the rapid induction of macrophage death caused by liposome injection. Alternatively, these data would support the suggestion that the mechanism by which macrophages are removed is responsible for the negative impacts on testicular function (Sauter *et al*., 2014) and highlights the need for further study in this area.

In summary, these ablation studies suggest that macrophages play an essential role in the development of the adult Leydig cell population and are also important for support of steroidogenesis in adulthood (Hutson, 1998), but have little direct impact on the seminiferous tubule.

## Sertoli Cells

Sertoli cell differentiation following SRY/SOX9 cascade activation is the first step of testis development. The important role that these cells play in orchestrating testicular differentiation (promotion of foetal Leydig cell and peritubular myoid cell development) and subsequent masculinisation of the foetus is well established (Svingen & Koopman, 2013). During foetal and early postnatal life there is a period of intensive Sertoli cell proliferation which ceases at puberty as the cells undergo a fundamental change in activity (Sharpe *et al*., 2003) (O'Shaughnessy, 2015). Inter‐Sertoli cell junctional complexes form to generate the BTB, which creates a stable microenvironment for germ cell development/maturation. In adulthood, Sertoli cells act to maintain spermatogenesis with each Sertoli cell able to support a fixed number of germ cells such that the numbers of spermatozoa produced is dependent upon total Sertoli cell number (Berndtson & Thompson, 1990). Overall, Sertoli cells are essential for testis differentiation but whether they remain the central drivers of testis development beyond this point or, indeed, whether they play a major role in adult testis function, beyond spermatogenesis, has remained unknown for many years. Clearly the answer to these questions could be provided by cell ablation studies although this has proven difficult with respect to Sertoli cells until recent technological developments. These recent studies have uncovered many previously unknown roles for Sertoli cells in testis development and function (Fig. 4).

It has been shown that Sertoli cells will undergo apoptosis postnatally in mice lacking Sertoli cell expression of Dicer, which is required for micro RNA and small interfering RNA biogenesis (Papaioannou *et al*., 2009; Kim *et al*., 2010). Sertoli cell apoptosis starts at about postnatal day 3 in these animals although some Sertoli cells survive into adulthood. There is massive germ cell apoptosis in these animals and by 6 months of age the architecture of the testis has degenerated with only rare tubules remaining among an apparent abundance of interstitial tissue (Papaioannou *et al*., 2009). This model is interesting in so far as the role of Dicer in the Sertoli cells is concerned but it does not meet all the criteria for a good experimental model of Sertoli cell ablation –that is it cannot be precisely controlled and it does not act quickly. Nevertheless, some of the testicular changes seen in these animals (tubule degeneration, germ cell loss) mirrors/confirms changes seen with a targeted approach to Sertoli cells ablation described below.

An alternative approach to Sertoli cell ablation has been to use cadmium, a heavy metal which will deplete the seminiferous tubules of their contents and, potentially, ablate the Sertoli cells (Shinohara *et al*., 2003) (Fig. 2). This also does not meet all the criteria of the ideal cell ablation method as the impact of cadmium is not restricted to one testicular cell type (Marettova *et al*., 2015) but the Sertoli cells appear to be the most vulnerable to cadmium exposure and even low doses have specific effects on the Sertoli cells (vacuolisation, cell contraction, detachment, inhibition of protein synthesis, apoptosis) (Clough *et al*., 1990; Zhang *et al*., 2010). Given its effect on the Sertoli cells, cadmium has been used to explore the role of the Sertoli cells in development of the spermatogonial stem cell niche. Transplantation of Sertoli cells into cadmium‐ and busulfan‐treated (Sertoli and germ cell depleted) seminiferous tubules (Shinohara *et al*., 2003) has permitted assessment of the potential of transplanted Sertoli cells to reconstitute a niche for spermatogonial stem cells and support active spermatogenesis. Using donor ROSA26‐l29 males, it was possible to evaluate the colonization ability of immature and adult Sertoli cells and results showed that immature dividing Sertoli cells are more able to effectively colonize the denuded seminiferous tubules and support recovery of spermatogenesis in recipient testes (Shinohara *et al*., 2003).

The recent development of new transgenic mouse models expressing cytotoxic genes or specific toxin receptors has opened the door to more refined methods of Sertoli cell ablation (Palmiter *et al*., 1987; Saito *et al*., 2001; Buch *et al*., 2005). Shinomura and colleagues (Shinomura *et al*., 2014) and our group (Rebourcet *et al*., 2014b) have independently applied similar approaches to drive the expression of diphtheria toxin A specifically in Sertoli cells from foetal life onwards, inducing Sertoli cell ablation from embryonic day 15. In addition, we have also combined an Amh‐Cre line (Lecureuil *et al*., 2002) and a iDTR line (Buch *et al*., 2005) to permit ablation of Sertoli cells via injection of DTX (Rebourcet *et al*., 2014a,b) (Fig. 2). The benefit of this inducible ablation model is that Sertoli cells can be ablated at different time points to assess temporal‐specific roles of this population not only in supporting testis development but also in adult function (Rebourcet *et al*., 2014a). These studies have emphasised the fundamental importance of Sertoli cells, revealing many previously undocumented roles that the cells play in the control of testis development and function (Rebourcet *et al*., 2014a,b; Shinomura *et al*., 2014).

As might be expected given the intricate relationship between Sertoli cells and germ cells, Sertoli cell ablation studies show that Sertoli cells are essential for germ cell survival, and that this is the case both in perinatal and in adult life. Injection of DTX leads to Sertoli cell death in the adult within 1 day and germ cell death follows rapidly, with no evidence of germ cells within the testis 10 days post DTX injection, including complete loss of mitotic spermatogonia (Rebourcet *et al*., 2014a; Shinomura *et al*., 2014). Only the reintroduction of immature Sertoli cells allows partial rescue of germ cell degeneration (Shinomura *et al*., 2014). These data confirm the crucial role Sertoli cells play in the development and maintenance of the germ stem cell niche.

More surprisingly, Sertoli cell ablation in foetal or perinatal life demonstrates that the tubular architecture is not fully established until puberty. Ablation of Sertoli cells either from 15.5 dpc or in neonatal life induces de‐differentiation of peritubular myoid cells, which no longer express smooth muscle markers. Recruitment of cells and re‐establishment of a myoid signature can be achieved if single cell testis aggregates are recombined in xenografts (Rebourcet *et al*., 2014b), demonstrating that Sertoli cells actively enforce a myoid fate on peritubular cells throughout foetal and pre‐pubertal development. In contrast, ablation of Sertoli cells during puberty (day 18) or in adulthood does not alter the tubular architecture suggesting that peritubular myoid cells fate is independent from Sertoli cells at this time, or that the joint deposition of the basement membrane by Sertoli and peritubular myoid cells is sufficient to stabilise the tubules and maintain myoid cell differentiation (Rebourcet *et al*., 2014b).

Our work has also revealed that Sertoli cells regulate the development of the adult Leydig cell population and are required for Leydig cell survival in adulthood. When Sertoli cells are ablated at postnatal day 2 or day 18 Leydig cell number in adulthood significantly decreases (70–90%), probably because of a reduction in the number of adult Leydig stem cells in the peritubular region. Perhaps more surprisingly, ablation of Sertoli cells in adulthood leads to a 75% decrease in LC number with the remaining cells concentrated in the sub‐capsular region and around the rete testis/tubuli recti. The nature of the factor or factors linking the Sertoli cells to Leydig cell development and survival remain uncertain but are of particular interest as they have the potential to regulate the size and activity of the adult Leydig cell population.

Finally, ablation of Sertoli cells illustrates the key role that the peritubular myoid cells play as a component of the blood–testis barrier. When the Sertoli cells are ablated in adulthood there is no barrier to infiltration of biotin into the tubules but only about 10% of tubules show evidence of immune cell penetration of the tubules (Rebourcet *et al*., 2014a). This shows that the peritubular myoid cells and underlying extracellular matrix form an effective barrier to macrophage invasion of the tubules. In addition, as peritubular myoid cells in the intact testis will normally act as a partial barrier to solute penetration of the tubule (Dym & Fawcett, 1970), failure to do this in the absence of Sertoli cells suggests that the function is Sertoli cell‐dependent.

## Conclusion

The development and application of these various models of cell‐specific ablation has significantly advanced our understanding of testis development and function. The dissection of the germ stem cell niche, the assignment of cell‐specific gene function, interactions between germ cells and somatic cells and the exploitation for transplantation studies have all benefitted from cell ablation approaches. Cell ablation has also had a significant impact on our understanding of Leydig cell development through identification of a postnatal stem cell population and then modelling the regeneration of this population. Exploring the role of the enigmatic macrophages, revealing their significant influence over Leydig cells and the interplay between the two cell types would not be possible without using cell ablation techniques. Finally, and most recently, identifying the previously unknown roles Sertoli cells play in germ cell support, peritubular myoid cell development and Leydig cell development and lifelong support have opened new avenues of research which can now be explored using the more conventional tool‐kit of the research scientist. With these studies describing the effects of Sertoli cell ablation, the remaining major cell type to be pursued in the testis is the peritubular myoid cell. It may prove difficult to target these cells specifically because of their similarity to smooth muscle cells but the effort is likely to be worthwhile as a number of recent studies have highlighted the importance of the peritubular myoid cells to testis function (Welsh *et al*., 2009, 2012; Nurmio *et al*., 2012; Qian *et al*., 2013; Chen *et al*., 2014). Other studies of interest would be the development of an inducible Leydig cell ablation model in the mouse to combine with the available gene knockout models and another testis macrophage ablation model to complement the earlier liposome studies.

While it is likely that specific gene ablation studies and other techniques will answer many of the remaining questions in testis biology, it can be argued that the types of cell ablation studies described here are essential precursors which lay the foundation for further advances in the field. Cell ablation as a technique lacks subtlety, but when used judiciously, it supports great leaps in understanding not possible via other approaches; as such it represents an essential component in the researchers’ tool‐kit, and should be viewed as a complement to the more mainstream approaches to advancing our understanding of testis biology.
